# Impact of Free and Nanoencapsulated *Echinophora platyloba* Essential Oil on the Physicochemical, Microbial, and Sensory Properties of Siahmazgi Cheese

**DOI:** 10.1002/fsn3.70514

**Published:** 2025-07-18

**Authors:** Nasim Hoseini, Alireza Shahab Lavasani, Marjaneh Sedaghati

**Affiliations:** ^1^ Department of Food Science and Technology, Faculty of Biological Sciences, North Tehran Branch Islamic Azad University Tehran Iran; ^2^ Department of Food Science and Technology, VaP.C. Islamic Azad University Tehran Iran

**Keywords:** cheese, microbial properties, nanocapsulation, physicochemical properties, sensory properties

## Abstract

This research investigated the effects of incorporating *Echinophora platyloba* essential oil (EPEO) on the qualitative and sensory characteristics of Siahmazgi cheese, a traditional Iranian cheese prepared from ewe's milk or a combination of ewe and goat's milk. This study analyzed various concentrations of EPEO, namely 0, 0.1%, 0.3%, and 0.5% (w/w), in both free and nanoencapsulated forms over 90 days. Initially, the nanoemulsion of EPEO was characterized by examining zeta potentials (ZPs) and microstructure. The resulting nanoemulsion capsules exhibited a spherical shape, with ZPs ranging from −0.74 ± 0.04 mV to −0.42 ± 0.05 mV. Subsequently, the influence of EPEO on Siahmazgi cheese was assessed through an analysis of its textural properties, including hardness, adhesiveness, and cohesiveness, as well as its chemical composition (specifically salt content), microbiological parameters (total bacterial counts, mesophilic bacterial counts, molds, and yeasts), and sensory characteristics (odor, taste, texture, and overall acceptability). The findings indicated that EPEO did not significantly alter the cheese's hardness but increased adhesiveness and decreased cohesiveness. Over time, both hardness and adhesiveness exhibited an upward trend, whereas cohesiveness diminished, all while maintaining a stable salt content. The minimum inhibitory concentrations (MICs) and minimum bactericidal concentrations (MBCs) were found to be 3.5 and 5 mg/mL against *Salmonella* and *Escherichia coli* , respectively. Furthermore, EPEO effectively reduced the counts of molds, yeasts, and total mesophilic bacteria in the treated cheese samples and improved flavor and overall acceptability, with the highest sensory ratings observed for the 0.3% (w/w) nanoencapsulated treatment over the 90‐day observation period. In summary, the incorporation of EPEO demonstrates significant potential as a natural additive for enhancing the qualitative and sensory attributes of Siahmazgi cheese.

## Graphical abstract

This study evaluated the impact of free and nanoencapsulated *Echinophora platyloba* essential oil on the physicochemical, microbial, and sensory properties of Siahmazgi cheese. Nanoemulsions showed spherical morphology and stable zeta potentials. The 0.3% nanoencapsulated treatment improved microbial safety, reduced molds and bacteria, and maintained favorable texture and flavor during 90 days. These findings highlight the potential of nanoencapsulated EPEO as a natural preservative to enhance traditional dairy products.

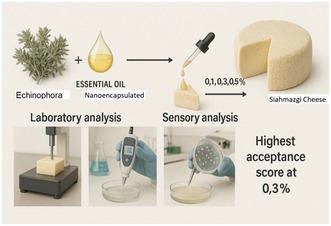

## 1 Introduction

Siahmazgi cheese is a traditional Iranian dairy product produced from ewe's milk or a combination of ewe and goat's milk. The aging process of this cheese occurs under specific conditions within sheepskin in Siahmazgi village, located in the suburbs of Rasht, Gilan province, Iran. This cheese is distinguished by its firm texture, pea‐sized holes, yellowish appearance, and a distinct fermented flavor (Erfanpoor et al. 2024). There has been a significant shift in consumer preferences in recent years, showing a growing aversion to synthetic chemical preservatives. This trend has sparked increased interest in natural alternatives that can help extend shelf life, enhance nutritional value, and improve sensory qualities in food products. As a result, both food industry stakeholders and academic researchers are increasingly focusing on utilizing active compounds derived from plant and fruit by‐products (Busetta et al. 2022). There is a growing consumer demand for products that emphasize quality, food safety, sensory appeal, and nutritional benefits. In particular, there is significant interest in cheese produced using traditional methods, as the sensory characteristics of this cheese often differ from those made through industrial processes (Bezerra et al. 2023). Currently, the industry is directed toward developing new products that utilize fewer chemical additives, including preservatives. This evolving landscape has prompted the food sector to seek alternative compounds that can aid in maintaining the stability of final products against microorganisms that can cause spoilage and foodborne illnesses (Bezerra et al. 2023).

Cheeses, particularly fresh varieties, are vulnerable to biochemical processes such as lipolysis and proteolysis. Their high water activity makes them susceptible to oxidation and microbiological deterioration, adversely affecting their shelf life (Sánchez‐Zamora et al. 2022). Essential oils (EOs) and extracts have gained considerable attention recently due to their natural origins and applicability in food products. These oils are best recognized for their low toxicity and strong antimicrobial and antioxidant properties (Hossen et al. 2024).

EOs consist of complex mixtures of various chemical compounds, many of which are classified as generally recognized as safe (GRAS). They possess noteworthy antioxidant and antimicrobial efficacy, making them promising candidates for biopreservatives to prevent spoilage and extend product shelf life (Dakhili et al. 2024; Mohammadi et al. 2023). However, their effectiveness can be limited by potential interactions with other components in the food matrix, high volatility, oxidation, and substantial changes in flavor and color within products. To address these challenges, EOs and extracts can be encapsulated within various delivery systems before incorporation into food items (Hossen et al. 2024).

*Echinophora platyloba* DC (*E. platyloba*) is an endemic plant in Iran known for its considerable medicinal properties, which have been extensively studied. This plant and its *Echinophora platyloba* essential oil (EPEO) are valued for their antimicrobial, antifungal, and antiseptic properties. In traditional practices, *E. platyloba* is referred to as “Khosharuz” or “Khusharizeh,” with its fresh and dried aerial parts commonly used as flavoring agents in a variety of foods, especially dairy products (Kebriti et al. 2025). To the best of our knowledge, this is the first study investigating the effects of free and nanoencapsulated EPEO on the physicochemical, microbial, and sensory properties of Siahmazgi cheese. The current research aims to produce Siahmazgi cheese using different concentrations of free and nano‐encapsulated EPEO while assessing their effects on the cheese's qualitative, microbial, and sensory properties.

## 2 Materials and Methods

### 2.1 Material Collection

*E. platyloba* was collected at the flowering stage from diverse regions within Gilan Province. The agro‐climatic conditions of the *E. platyloba* harvested region are characterized by a humid subtropical climate, a mean annual temperature of 16°C and an average annual rainfall of 1100 mm. Initially, the specimens underwent washing with water to eliminate surface dust. Following this preliminary washing, the aerial parts of the plant were carefully separated and completely dried in a shaded environment. After drying, the plant material was subsequently ground into a fine powder using an electric grinder. The EO was extracted using a Clevenger apparatus through water distillation (Kebriti et al. 2025). The extracted EOs were dried over anhydrous sodium sulfate (Merck, CAS No. 7757‐82‐6, analytical grade, Purity: ≥ 99.0%) and stored at a temperature of −20°C ± 2°C until needed for further analysis. It was contained in dark vials to preserve the integrity of the extracted EO to prevent light exposure and subsequent alterations in its properties. Additionally, ewe milk was sourced from the Gachsar region of Karaj.

### 2.2 Preparation of Nanoemulsion

**FIGURE 1:**
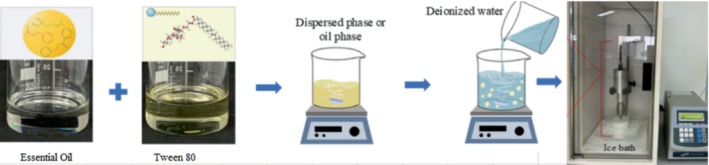
Steps for preparing the nanoemulsion used in the formulation of Siahmazgi cheese.

The EO derived from *E. platyloba* was initially combined in equal proportions with Tween 80 (Merck, CAS No. 9005‐65‐6, grade of food grade, Purity: ≥ 99.0%), which served as a surfactant, using a magnetic stirrer. Subsequently, distilled water was slightly acidified with 0.3% (w/w) citric acid (Sigma‐Aldrich, CAS No. 77‐92‐9, grade of ACS, Purity: ≥ 99.0%) to create the aqueous phase. The oil and surfactant mixture was then gradually introduced into the acidified water while continuously stirring, resulting in an emulsified pre‐mixture (Figure 1). This pre‐mixture was later subjected to ultrasonic waves (Hielscher, Teltow, Germany) for 20 min in an ultrasonic bath operating at a power of 100 W and a frequency of 40 kHz, which helped to reduce particle size and produce a nanoemulsion (Kebriti et al. 2025).

### 2.3 Characterization of Nanoemulsion

The zeta potential (ZP) of the prepared emulsions was measured at a temperature of 25°C ± 2°C and a 90° angle using a dynamic light scattering instrument, specifically the Horiba SZ‐100‐Z (Horiba, Japan), as reported by (Kebriti et al. 2025).

The morphology of the nanoemulsion droplets was examined using a Transmission Electron Microscope (TEM) (Zeiss, model: CEM902A) with a Philips EM 208 S instrument. A single drop of the nanoemulsion was placed onto a copper grid and stained with a 2% (w/v) phosphotungstic acid solution for 1 min. The grid was subsequently observed at an acceleration voltage of 10 kV.

### 2.4 Cheese Production

**FIGURE 2:**
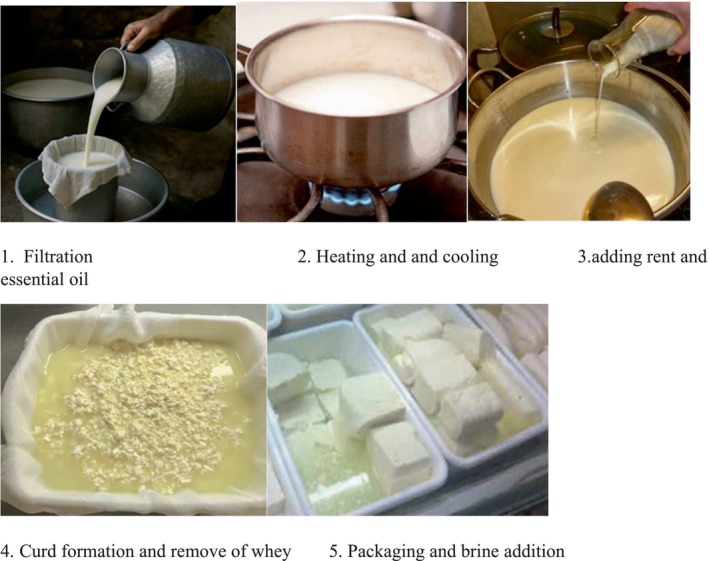
Cheese production steps.

Ewe milk was heated to 80°C for 5 min to eliminate bacteria and contaminants. It was then divided into seven portions for various treatments, each containing 10 kg of milk. After cooling to 40°C ± 2°C, 5 g of industrial enzyme rennet per liter was added, initiating the curdling process within 1 to 2 min. EOs were incorporated at concentrations of 0.1%, 0.3%, and 0.5% (w/w) (both free and encapsulated) once signs of curd formation appeared (Figure 2). Following curd formation, the cheese was pressed to remove the whey, packaged in polystyrene containers, and treated with 10% (w/v) brine before refrigerating for 3 months at 4°C ± 1°C (Pirsaraii et al. 2021). Cheese samples were analyzed for physicochemical, microbiological, and sensory properties during storage on days 1, 30, 60, and 90.

### 2.5 Texture Analysis

The textural properties of processed cheese were analyzed using the Texture Profile Analyzer (TPA) (QTS25, FARNELL, England). To minimize surface effects, samples for the texture profile analysis were collected from the center of the cheese block. The assessment was performed with the TA 17 probe, featuring 30 and 25 mm diameters, and operated at a crosshead speed of 1 mm/s with a penetration distance of 10 mm. Hardness, cohesiveness, and adhesiveness were evaluated in triplicate (Florczuk et al. 2022).

### 2.6 Salt

The determination of sodium chloride was conducted using the AOAC method 971.19.

### 2.7 Determination of MIC and MBC Values

The minimum inhibitory concentration (MIC) of EPEO was evaluated using a modified broth microdilution method (Mohammadi et al. 2023). The EO was diluted in Tryptic Soy Broth (TSB) (Merck, Germany) supplemented with 0.1% (w/v) dimethyl sulfoxide (DMSO) (Merck, CAS No. 67‐68‐5, grade of ACS, Purity: ≥ 99.0%) to enhance solubility. Concentrations ranging from 0.05% to 1% (w/w) of the EO were prepared in 96‐well microtiter plates, each containing 100 μL of a fresh cell suspension (5 × 10^5^ cfu/mL). Positive controls comprised the inoculated growth medium without EO, while negative controls contained only the EO in a sterile medium. The plates were incubated aerobically at 37°C ± 1°C for 24 h. The MIC was defined as the lowest concentration at which no visible bacterial growth was observed. To determine the minimum bactericidal concentration (MBC), 5 μL from wells exhibiting no growth in the MIC test was streaked onto blood agar (Merck, Germany) and further incubated at 37°C ± 1°C for 24 h. The MBC was identified as the lowest concentration that reduced the initial growth by 99.9% or more. The evaluations of MIC and MBC were performed for each of the two bacterial strains tested (Mohammadi et al. 2023).

### 2.8 Microbiological Analyses

Cheese samples (10 g) were homogenized in 90 mL of a 2% (w/v) Trisodium citrate solution using a stomacher Bag‐Mixer (Model: Bag Tech S‐400) for 2 min, followed by a 1:10 serial dilution. Total mesophilic microorganisms (TMM) were enumerated using skim milk plate count agar and incubated at 30°C ± 1°C for 72 h. Coliforms were detected on violet red bile lactose agar (VRBLA) (Merck, Germany), incubating at 30°C ± 1°C for 24 h. Yeasts were quantified using Dichloran Rose Bengal Chloramphenicol (DRBC) agar (Merck, Germany), incubating at 25°C ± 1°C for 96 h. The identification of *Escherichia coli* and *Salmonella* spp. was performed on Hektoen enteric agar (HEA) (Merck, Germany), incubated at 37°C ± 1°C for 24 h (Maniaci et al. 2024).

### 2.9 Sensory Analysis

Cheese treatments were diced into 2 × 2 × 2 cm^3^ cubes and arranged on plastic plates, each labeled with a randomly assigned 3‐digit code. A group of 10 trained panelists conducted the sensory evaluation of these cheese samples. They assessed various sensory attributes, including flavor, color, texture, and overall acceptability, using a 5‐point hedonic scale that ranged from 1 (very unpleasant) to 5 (very pleasant) (Norouzbeigi et al. 2021; Yekta et al. 2024).

### 2.10 Statistical Analysis

The influence of EPEO on the quality characteristics of the samples was thoroughly analyzed using one‐way ANOVA with SPSS software version 15. Duncan's Multiple Range Test was utilized to evaluate the differences between the means. A significance level of *p* < 0.05 was set for all assessments. The findings are displayed as mean values and standard deviations in the relevant tables and figures.

## 3 Results and Discussion

### 3.1 Nanoemulsion Characterization

**FIGURE 3:**
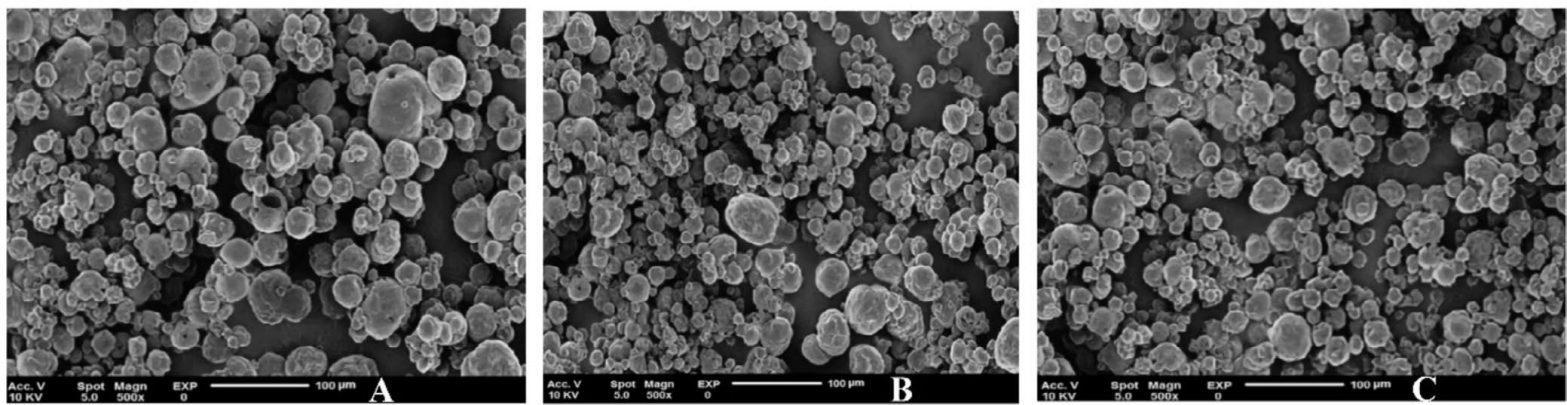
TEM images of 0.1% (A), 0.3% (B), and 0.5% (C) encapsulated EPEO.

ZP is a vital indicator of the physical stability of emulsions during storage (Kebriti et al. 2025). More to the point, the magnitude of the ZP indicates the extent of electrostatic repulsion between similarly charged molecules or particles within the emulsion. Furthermore, the van der Waals forces contribute to the aggregation of oil droplets in the emulsion, which results in a more negative ZP (Liu et al. 2020). The observed ZP of the emulsions ranged from −0.74 ± 0.04 mV to −0.42 ± 0.05 mV. Figure 3 illustrates TEM images of encapsulated EPEO at concentrations of 0.1%, 0.3%, and 0.5% (w/w). These images demonstrate that the resulting capsules are spherical and vary in size, with smaller spherical nanocapsules situated among larger ones, resulting in an irregular arrangement. In a similar investigation, the higher antimicrobial properties of curcumin nanoemulsions were correlated with a higher surface‐to‐volume ratio, surface charge, and hydrophobicity (Ibrahim El‐Sayed and Shalaby 2021).

### 3.2 Textural Properties

**FIGURE 4:**
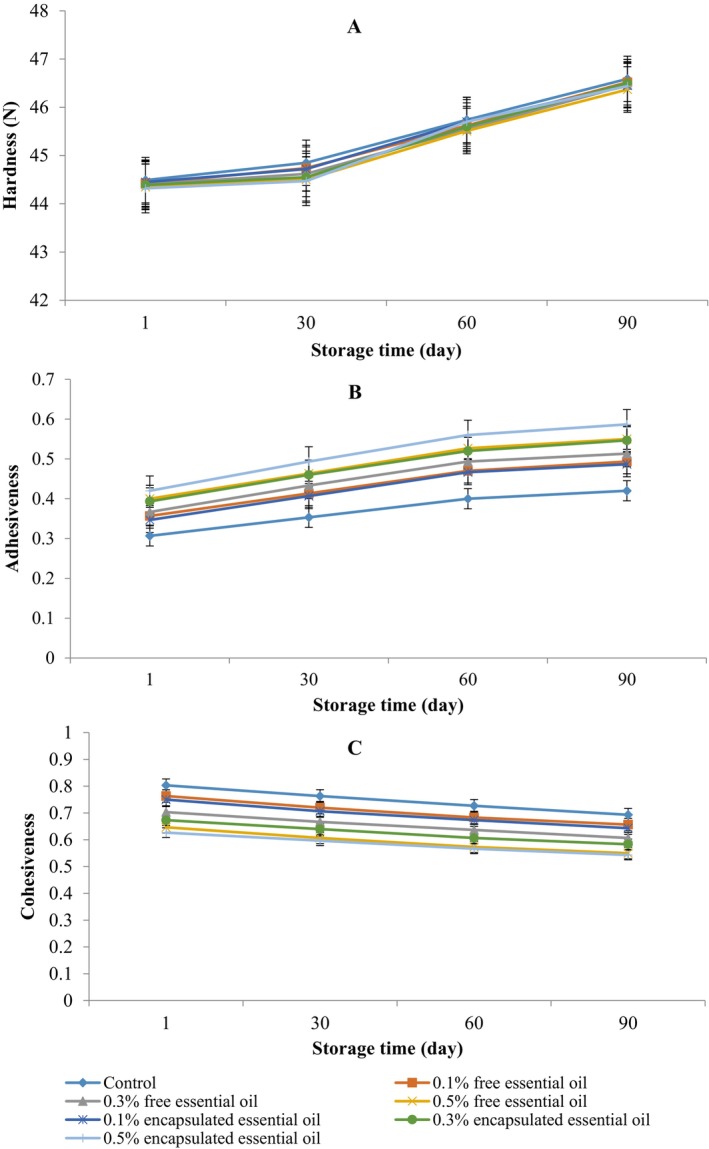
Changes in textural properties of Siahmazgi cheese during the storage period: Hardness (A), adhesiveness (B), and cohesiveness (C). Data are presented as mean ± standard deviation.

The results indicate no significant difference between the treatments using free and encapsulated EOs in terms of changes in hardness (*p* > 0.05). Over the 90‐day storage period, the hardness of the cheese samples increased, as illustrated in Figure 4A. This observation aligns with the findings of who found no significant difference in texture between the control cheese and the cheese containing encapsulated particles. However, El‐Sayed and Thanaa (2021) observed a decrementation in hardness values of processed cheese incorporated with 2.5% and 5% (w/w) curcumin nanoemulsions compared to the plain treatment. Moreover, incorporating microcapsules and nanoemulsions of EOs into cheese affects its hardness by modifying the protein–fat network structure (Bagale et al. 2023). Notably, microcapsules significantly alter the cheese's microstructure due to their larger size than nanoemulsions. This alteration impedes the formation of a uniform network among the components, impacting the overall texture (Pérez‐Soto et al. 2021). In our study, we incorporated lower concentrations of EPEO (up to 0.5% (w/w)) compared to the EO concentrations used in recent investigations (2.5% to 5% (w/w)). Consequently, the nanoemulsion had a minimal effect on the hardness values of cheese treatments relative to the control treatment (*p* > 0.05).

Overall, the hardness of all cheese treatments significantly increased during the 3‐month study period (*p* < 0.05). One key reason for the increased firmness of the cheese is the more significant loss of water from the cheese matrix and an increase in the percentage of dry matter starting from day 30 of storage. The findings suggest that the effect of proteolysis is less significant than the reduction in moisture; consequently, the strength of the texture, influenced by moisture loss, has a considerable effect on both the texture and firmness of the cheese (Cai et al. 2024).

EPEO‐incorporated cheese treatment displayed increased adhesiveness and reduced cohesiveness compared to the control cheese (Figure 4B,C).

Throughout the storage period, all cheese samples exhibited a consistent trend regarding cohesiveness, which declined as adhesiveness increased. The gradual increase in cheese hardness can be attributed to moisture loss over time, as supported by Cai et al. (2024). Adhesiveness and cohesiveness were also affected, likely due to proteolytic degradation of casein and the formation of stronger protein networks (Bagale et al. 2023).

### 3.3 Salt

**FIGURE 5:**
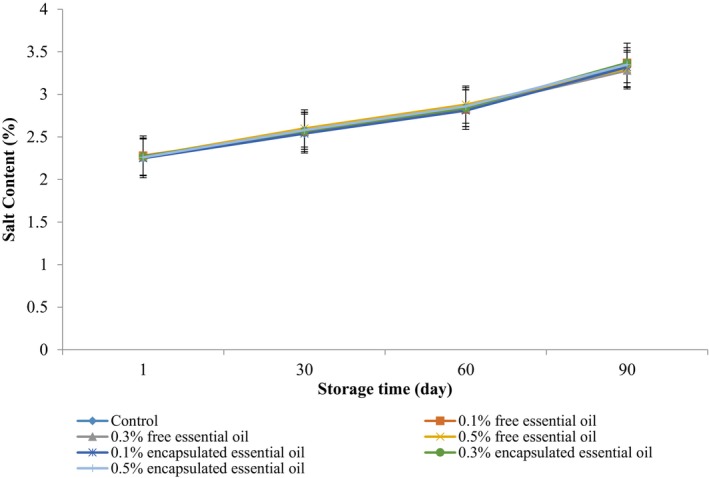
Changes in the salt content of Siahmazgi cheese during the storage period. Data are presented as mean ± standard deviation.

The salt content significantly influences cheese's shape, texture, flavor, and quality. Figure 5 revealed that incorporating EPEO did not significantly alter the salt content in the cheese (*p* > 0.05). Nevertheless, there was a notable increase in salt content during the storage period (*p* < 0.05). According to Eljagmani and Altuner (2020), the length of storage has a significant effect on the cheese's salt content (*p* < 0.05).

### 3.4 Microbial Characteristics

The results revealed that the MIC and MBC for *Salmonella* and *E. coli* were determined to be 3.5 and 5 mg/mL, respectively. Sharafati‐chaleshtori et al. (2012) found that both aqueous and alcoholic extracts of *E. platyloba* exhibited antimicrobial properties against Gram‐negative and Gram‐positive bacteria. Additionally, Bazvandi et al. (2017) highlighted that these extracts demonstrated more significant inhibitory activity against Gram‐positive bacteria than Gram‐negative bacteria, which can be attributed to differences in their cell wall structures.

**FIGURE 6:**
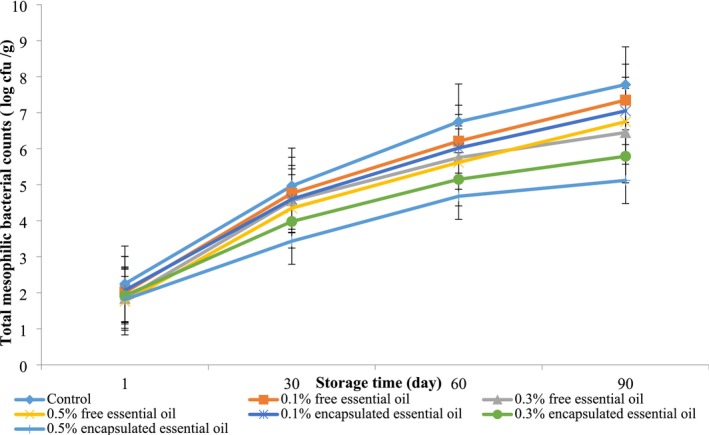
The effects of EPEO on total mesophilic counts of Siahmazgi cheese during storage. Data are presented as mean ± standard deviation.

**FIGURE 7:**
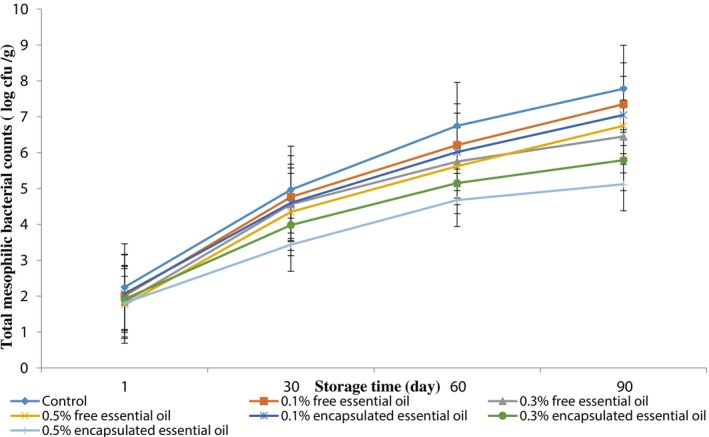
The effects of EPEO on yeasts and molds of Siahmazgi cheese during storage. Data are presented as mean ± standard deviation.

Our findings indicated no *Salmonella* or *E. coli* growth in any treatments (Table 1). A significant difference was found between the treatments containing free and encapsulated EOs for yeast, mold, and total mesophilic bacterial counts (*p* < 0.05). Over the storage period, there was an increase in yeast and mold counts and total mesophilic bacterial counts across all treatments. Furthermore, the results indicated that a higher EPEO concentration significantly reduced yeast, mold, and total aerobic bacterial counts (*p* < 0.05), as shown in Figures 6 and 7.

**TABLE 1:** Microbiological analysis of Siahmazgi cheese during refrigerated storage.

Treatments	Days			
	1	30	60	90
*E. coli* (log cfu/g)				
Control	N.D.	N.D.	N.D.	N.D.
0.1% free essential oil	N.D.	N.D.	N.D.	N.D.
0.3% free essential oil	N.D.	N.D.	N.D.	N.D.
0.5% free essential oil	N.D.	N.D.	N.D.	N.D.
0.1% encapsulated essential oil	N.D.	N.D.	N.D.	N.D.
0.3% encapsulated essential oil	N.D.	N.D.	N.D.	N.D.
0.5% encapsulated essential oil	N.D.	N.D.	N.D.	N.D.
*Salmonella* (log cfu/g)				
Control	N.D.	N.D.	N.D.	N.D.
0.1% free essential oil	N.D.	N.D.	N.D.	N.D.
0.3% free essential oil	N.D.	N.D.	N.D.	N.D.
0.5% free essential oil	N.D.	N.D.	N.D.	N.D.
0.1% encapsulated essential oil	N.D.	N.D.	N.D.	N.D.
0.3% encapsulated essential oil	N.D.	N.D.	N.D.	N.D.
0.5% encapsulated essential oil	N.D.	N.D.	N.D.	N.D.

Abbreviation: N.D., not detected.

The results indicate that the control treatment exhibited the highest counts of yeasts, molds, and total aerobic bacteria, while the samples containing 0.5% (w/w) encapsulated EPEO demonstrated the lowest counts of total aerobic bacteria, yeasts, and molds. This difference was statistically significant (*p* < 0.05). The study illustrated that incorporating free and encapsulated EPEO declined bacterial, yeasts, and mold growth throughout the storage period.

Entezari et al. (2009) reported that the methanolic extract of *E. platyloba* possesses antimicrobial properties against *Staphylococcus aureus* and *Pseudomonas aeruginosa* . According to Bazvandi et al. (2017), the antimicrobial efficacy of the methanolic extract can be attributed to specific active compounds, such as stigmasterol and sitosterol. The nanoemulsion technique improves the dispersion of EOs in aqueous formulations, enhances their chemical stability during storage, minimizes sensory changes, and may even boost antimicrobial properties. The reduction in particle size increases the surface area available for contact, thereby enhancing antimicrobial activity (Bedoya‐Serna et al. 2018).

Ehsani et al. (2016) found that EPEO at a concentration of 0.5% (w/w) significantly lowered the count of aerobic bacteria in pasteurized cream (*p* < 0.05). Moreover, Nasiri et al. (2024) indicated that *E. platyloba* contains phenolic compounds such as carvacrol, thymol, and coumarin, which may be effective against *Lactobacillus* bacteria.

### 3.5 Sensory Properties

The spoilage of dairy products is characterized by changes in flavor, color, appearance, and texture (Barros et al. 2020). Previous studies have demonstrated that the administration of EOs in cheese enhances its aroma, taste, and overall acceptability, although it does not significantly affect the texture (Christaki et al. 2021; Falih et al. 2024). During the storage period, the sensory property ratings tended to decline; however, samples containing encapsulated EPEO showed a minor reduction (Table 2).

**TABLE 2:** Sensory properties of Siahmazgi cheese during refrigerated storage. Data are presented as mean ± standard deviation.

Treatments	Days			
	1	30	60	90
Odor				
Control	4.3 ± 0.48^Ab^	4.1 ± 0.31^Ab^	3.6 ± 0.51^Bb^	3.3 ± 0.48^Bd^
0.1% free essential oil	4.5 ± 0.52^Aab^	4.3 ± 0.48^Aab^	3.8 ± 0.42^Bab^	3.6 ± 0.51^Bbc^
0.3% free essential oil	4.9 ± 0.31^Aa^	4.7 ± 0.48^Aa^	4.3 ± 0.48^Ba^	4.1 ± 0.31^Ba^
0.5% free essential oil	4.0 ± 0.51^Aab^	4.2 ± 0.63^ABab^	4.1 ± 0.31^ABab^	4.0 ± 0.47^Bab^
0.1% encapsulated essential oil	4.4 ± 0.69^Aab^	4.1 ± 0.73^ABb^	3.8 ± 0.63^ABb^	3.5 ± 0.52^Bd^
0.3% encapsulated essential oil	4.8 ± 0.42^Aab^	4.6 ± 0.51^ABab^	4.4 ± 0.51^ABa^	4.2 ± 0.42^Ba^
0.5% encapsulated essential oil	4.6 ± 0.51^Aab^	4.4 ± 0.51^ABab^	4.2 ± 0.42^ABab^	4.0 ± 0.00^Bab^
Taste				
Control	3.9 ± 0.31^Ab^	3.7 ± 0.48^ABb^	3.4 ± 0.51^BCc^	3.1 ± 0.17^Cc^
0.1% free essential oil	4.4 ± 0.69^Aab^	4.0 ± 0.66^ABb^	3.8 ± 0.42^ABbc^	3.4 ± 0.22^Bbc^
0.3% free essential oil	4.9 ± 0.31^Aa^	4.7 ± 0.48^ABa^	4.4 ± 0.51^BCa^	4.2 ± 0.13^Ca^
0.5% free essential oil	4.4 ± 0.69^Aab^	4.2 ± 0.63^Aab^	3.9 ± 0.56^Ab^	4.0 ± 0.25^Aab^
0.1% encapsulated essential oil	4.4 ± 0.69^Aab^	4.0 ± 0.66^ABb^	3.9 ± 0.56^ABb^	3.5 ± 0.16^Bbc^
0.3% encapsulated essential oil	4.8 ± 0.31^Aa^	4.6 ± 0.51^ABa^	4.4 ± 051^ABa^	4.2 ± 0.20^Ba^
0.5% encapsulated essential oil	4.4 ± 0.69^Aab^	4.2 ± 0.63^Aab^	4.1 ± 0.31^Aab^	4.0 ± 0.21^Aab^
Texture				
Control	4.3 ± 0.67^Aa^	4.3 ± 0.48^Aa^	4.4 ± 0.51^Aa^	4.5 ± 0.52^Aa^
0.1% free essential oil	4.3 ± 0.48^Aa^	4.3 ± 0.48^Aa^	4.3 ± 0.48^Aa^	4.5 ± 0.52^Aa^
0.3% free essential oil	4.2 ± 0.42^Aa^	4.3 ± 0.67^Aa^	4.4 ± 0.51^Aa^	4.5 ± 0.52^Aa^
0.5% free essential oil	4.2 ± 0.63^Aa^	4.3 ± 0.48^Aa^	4.3 ± 0.67^Aa^	4.3 ± 0.67^Aa^
0.1% encapsulated essential oil	4.3 ± 0.67^Aa^	4.3 ± 0.48^Aa^	4.4 ± 0.69^Aa^	4.3 ± 0.67^Aa^
0.3% encapsulated essential oil	4.4 ± 0.51^Aa^	4.4 ± 0.51^Aa^	4.4 ± 0.51^Aa^	4.5 ± 0.52^Aa^
0.5% encapsulated essential oil	4.3 ± 0.67^Aa^	4.3 ± 0.67^Aa^	4.2 ± 0.42^Aa^	4.4 ± 0.51^Aa^
Overall acceptance				
Control	4.3 ± 0.48^Ab^	3.9 ± 0.56^ABb^	3.6 ± 0.51^Bc^	3.1 ± 0.56^Cd^
0.1% free essential oil	4.4 ± 0.69^Aab^	4.0 ± 0.66^ABb^	3.8 ± 0.42^ABb^	3.4 ± 0.69^Bcd^
0.3% free essential oil	4.9 ± 0.31^Aa^	4.7 ± 0.48^Aa^	4.4 ± 0.51^Ba^	4.10 ± 0.56^Ba^
0.5% free essential oil	4.5 ± 0.52^Aab^	4.2 ± 0.63^ABab^	4.1 ± 0.87^Bab^	3.9 ± 0.56^Babc^
0.1% encapsulated essential oil	4.4 ± 0.69^Aab^	4.0 ± 0.66^ABb^	3.9 ± 0.56^ABab^	3.5 ± 0.52^Bbcd^
0.3% encapsulated essential oil	4.9 ± 0.31^Aa^	4.6 ± 0.51^ABa^	4.4 ± 0.51^Ba^	4.2 ± 0.63^Ba^
0.5% encapsulated essential oil	4.6 ± 0.51^Aab^	4.2 ± 0.63^ABab^	4.1 ± 0.31^ABab^	4.0 ± 0.66^Bab^

*Note:* Lowercase letters in each row and uppercase letters in each column are not significantly different at the 5% probability level.

In general, treatments that contained either high or low concentrations of EPEO received lower sensory scores, with the formulation containing 0.3% (w/w) encapsulated EPEO being recognized as the most favored option. Sensory analysis indicated that while the use of EPEO did not drastically alter the overall acceptability, nanoencapsulation contributed to better retention of aroma and flavor over the storage period. These findings are in line with those of Christaki et al. (2021) and Azarashkan et al. (2022), who emphasized that encapsulated systems preserve volatile flavor compounds more efficiently. The implementation of encapsulated EPEO led to enhanced aroma and taste retention compared to free EOs during storage.

Bakhshi et al. (2020) revealed that the incorporation of basil EO improved the texture consistency of cheese throughout the storage period, particularly in samples with elevated EO concentrations; however, it had little effect on the appearance of soft cheese (Bakhshi et al. 2020). Ehsani et al. (2016) found that pasteurized cream samples enriched with sweet basil EO demonstrated better sensory properties than the control, with the 0.1% (w/w) EO treatment achieving the highest aroma rating.

Azarashkan et al. (2022) noted that the sensory scores of cheese decreased during storage due to oxidative, chemical, and microbial processes. The improvement in sensory characteristics of encapsulated samples is attributed to the flavor‐masking properties of the EO (Azarashkan et al. 2022). Hamdy and Hafaz (2018) illustrated that adding rosemary, basil, and thyme favorably impacted the sensory qualities of ricotta cheese despite a slight reduction in overall acceptance during the storage period.

The rise in free amino acids and free fatty acids represents critical compounds that enhance the aroma and flavor of cheeses. The proteolytic activities and the proteins present in cheese during aging result in the formation of free amino acids and small peptides, which are influenced by rennet and microbial peptidases and are recognized as key contributors to cheese aroma and flavor. Additionally, volatile fatty acids produced through the lipolysis of milk fats during the cheese ripening process also play a significant role in the aroma and flavor profile, with their concentrations gradually increasing throughout the aging process (Chen et al. 2024).

## 4 Conclusion

This study investigated the impact of various concentrations (0% to 0.5% w/w) of free and nanoencapsulated EPEO on the physicochemical, microbial, and sensory properties of traditional Siahmazgi cheese. The nanoemulsion system exhibited spherical nanocapsules with moderate ZP values, suggesting structural stability. Although the inclusion of EPEO did not significantly influence the texture in terms of hardness, it notably altered adhesiveness and cohesiveness, especially during storage.

Microbial analysis demonstrated that EPEO exhibited strong antimicrobial activity against *Salmonella* and *E. coli* , with nanoencapsulation further enhancing its efficacy. The 0.5% w/w nanoencapsulated treatment significantly reduced mold, yeast, and total mesophilic counts, maintaining better microbial stability compared to the control samples. Sensory evaluation revealed that the 0.3% w/w nanoencapsulated EPEO treatment achieved the highest acceptability scores for aroma and taste.

Overall, the findings confirm that nanoencapsulated EPEO can serve as a promising natural preservative in traditional cheese products, contributing to improved microbial safety and sensory quality during extended storage. These results support the broader application of nanoencapsulated plant‐based antimicrobials in functional and clean‐label dairy systems.

## Author Contributions

**Nasim Hoseini:** investigation (equal), writing – original draft (equal). **Alireza Shahab Lavasani:** conceptualization (equal), methodology (equal), supervision (equal). **Marjaneh Sedaghati:** writing – review and editing (equal).

## Ethics Statement

The authors have nothing to report.

## Conflicts of Interest

The authors declare no conflicts of interest.

## Data Availability

The data used to support the findings of this study are available from the corresponding author upon request.
